# DeVa (Decay Variance): A Novel Score Calculated via Postprocessing the Changes in Signal Intensity of an Intervertebral Disc in a T2* Multi‐Echo Magnetic Resonance Image Can Quantify Painful and Degenerate Lumbar Vertebral Discs

**DOI:** 10.1002/jsp2.70056

**Published:** 2025-03-06

**Authors:** Stone Sima, Alisha Sial, Suhani Sharma, Dheera Ananthakrishnan, Jeff Kuan, Ashish Diwan

**Affiliations:** ^1^ Spine Labs, Department of Orthopedic Surgery, St George and Sutherland Clinical School University of New South Wales Sydney Australia; ^2^ Spine Service, Department of Orthopaedic Surgery St George Hospital Sydney Australia; ^3^ Spinal Unit, Discipline of Orthopaedic Surgery School of Medicine, University of Adelaide Adelaide Australia; ^4^ Department of Orthopaedic Surgery Emory University Hospital Atlanta Georgia USA; ^5^ Kogarah Imaging Centre, Department of Radiology St George Hospital Sydney Australia

**Keywords:** decay variance, intervertebral disc degeneration, low back pain, novel MRI postprocessing, signal intensity

## Abstract

**Introduction:**

Low back pain (LBP), a global disability leader, is often linked to intervertebral disc (IVD) degeneration. Traditional diagnostics like T2‐weighted MRI provide qualitative but imprecise evaluations. A novel post‐processing MRI technique, Decay Variance (DeVa), has shown promise in differentiating degenerate from healthy discs in animal studies. DeVa quantifies IVD degeneration by analyzing variations in signal intensities within each voxel in a T2* 2D FLASH multi‐echo MRI sequence. This study aimed to validate DeVa clinically and explore its correlation with pain severity.

**Methods:**

A cross‐sectional study included 77 chronic LBP patients and 8 controls, who underwent T2‐weighted and T2* 2D FLASH MRI. DeVa scores (worst and sum of all discs) were recorded, alongside traditional assessments like disc bulge, stenosis, high‐intensity zones, and Pfirrmann grade. Pain severity was measured with a numerical rating scale. Statistical analyses included Pearson correlation, *t*‐tests, and Gardner‐Altman plots to evaluate relationships between DeVa scores, degeneration, and pain.

**Results:**

DeVa scores correlated strongly with Pfirrmann grade (*r* = 0.692, *p* < 0.001) and were significantly higher in discs with bulge, stenosis, or high‐intensity zones (*p* < 0.001). Moderate correlations were observed between worst DeVa scores (*r* = 0.296, *p* < 0.01), total DeVa scores (*r* = 0.323, *p* < 0.005) and pain severity. Patients with chronic LBP without severe degeneration (Pfirrmann ≤ 3 with no stenosis observable on standard MRI) had significantly higher worst (1.38 ± 0.26 vs. 1.10 ± 0.29, *p* < 0.005) and total (5.39 ± 0.75 vs. 4.65 ± 0.61, *p* < 0.0.1) DeVa scores compared to controls.

**Discussion:**

DeVa offers a quantitative, noninvasive approach to assessing IVD degeneration, showing strong correlations with disc health and pain. It demonstrates enhanced sensitivity over traditional MRI, enabling the identification of pain‐generating discs and informing personalized treatment strategies for chronic LBP. Further validation in larger populations is needed.

## Graphical abstract

The study introduces Decay Variance (DeVa), a novel MRI postprocessing technique to assess intervertebral disc degeneration. DeVa quantitatively correlates with disc health and pain, demonstrating greater sensitivity than traditional imaging. It shows potential for noninvasive diagnostics, aiding in the personalized management of chronic low back pain.

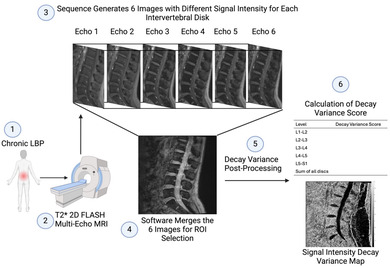

## 1 Introduction

Low back pain (LBP) is the largest cause of disease burden from disability worldwide, affecting over 619 million individuals in 2020, with that number expected to rise to 843 million in 2050 [1]. Degeneration of the lumbar intervertebral disc (IVD) is one putative cause of such nonspecific LBP, and 26%–42% of LBP has been attributed to the disc‐related causes [2, 3]. The IVD is a connective tissue structure connecting adjacent vertebral bodies. It is composed of an inner nucleus pulposus (NP) which is surrounded circumferentially by an outer annulus fibrosus (AF) and capped inferiorly and superiorly by a cartilaginous endplate (EP) attached to the subjacent and suprajacent vertebral body. Degeneration is a complex biochemical process consisting of a loss of cellularity, changes in signaling molecules, infiltration of proinflammatory cytokines, and dysregulation of extracellular matrix maintenance with dominance of catabolic processes [4, 5, 6]. Current literature has shown that IVD degeneration alone is associated with LBP and neuropathic pain clinically, and loss of disc height biomechanically [7, 8, 9, 10].

Despite this known knowledge, in clinical practice more than 90% of patients with LBP experience symptoms lacking a clear pathoanatomical cause that can be identified with methods of investigation that are currently available. Traditionally, IVD degeneration has been assessed using T2‐weighted magnetic resonance imaging (T2W MRI) with a qualitative grading scale described by Pfirrmann and colleagues [11]. Despite the ubiquity of this grading scale, their method demands extensive morphological knowledge of the IVD, rendering it predominantly utilized in research settings. Furthermore, there exists a questionable clinical utility due to the reliability of the scoring method and the requirement of the point‐of‐care clinician to undertake the job of generating that score.

The field of quantitative MRI in assessing degenerative IVD is advancing rapidly, with publications showing T2* quantitative relaxometry and T1‐rho to be correlated with IVD degeneration in pre‐clinical studies [12, 13]. However, these techniques are time‐consuming, with patients in the scanner for more than 30 min and hence costly. Furthermore, there is resistance from radiology practices due to a perceived lack of financial reward. Therefore, improved noninvasive and less‐intrusive imaging methods are needed for a better assessment of disc degeneration.

Our laboratory has developed a novel MRI postprocessing technique termed Decay Variance (DeVa), utilizing a T2* multi‐echo gradient echo (MR‐GRE) sequence. T2* is more sensitive than T2 to microstructural heterogeneity and biochemical changes, such as variations in hydration and glycosaminoglycan content, making it a superior metric for assessing early disc degeneration [14, 15]. While local field inhomogeneities affect T2*, the DeVa technique leverages these variations to capture biologically relevant tissue differences rather than treating them as artifacts. DeVa provides a more precise quantification of the heterogeneous composition of the IVD by analyzing the variance in decay times within a voxel from MRI data collected at multiple echo times. Initial in silico exploratory simulations demonstrated that elevated DeVa scores correlate with reduced concentrations of water and glycosaminoglycans—well‐established markers of disc health [16]. Based on these findings, we hypothesized that increasing IVD degeneration severity would result in higher DeVa scores. This hypothesis was tested using an animal model in which disc degeneration was induced via annular puncture in 25 New Zealand White rabbits. The study confirmed that DeVa more strongly correlated with the histological extent of degeneration compared to traditional qualitative grading methods and T2 mapping‐derived relaxation rates. Furthermore, DeVa calculation was less computationally intensive and more efficient [17]. The DeVa technique has been shown to distinguish between histologically degenerate and histologically healthy discs in animals, but validation on human subjects was needed prior to clinical implementation.

A subsequent first‐in‐human feasibility study (*n* = 8) demonstrated that the DeVa‐based scan was rapid (< 5 min), reproducible, and allowed for post‐processing, thereby reducing the burden on the radiographer. The results also indicated higher DeVa scores in patients experiencing severe pain compared to those with moderate pain [16].

Moving towards clinical implementation, this study serves as a clinical validation of the DeVa technique in the assessment of IVD health and its correlation with disease severity. As such, a single‐centre cross‐sectional cohort study was conducted with two aims. Firstly, the study aimed to determine the association between DeVa score and known lumbar degenerative radiological measurements on T2W MRI. Secondly, the study aimed to assess whether the DeVa technique can distinguish between patients suffering from LBP with disc degeneration as a component cause and those without LBP.

## 2 Methods

### 2.1 Study Design and Population

The study received approval from the University of New South Wales Human Research Ethics Panel (No. HC190571). All patients provided informed consent prior to enrolling in the study. A single‐center cross‐sectional cohort study was conducted from August 2020 to June 2024 involving patients with chronic LBP referred by primary physicians to a single Tertiary Spine Clinic in Kogarah, NSW. A comparative analysis between patients with chronic LBP and asymptomatic controls was also conducted. The inclusion and exclusion criteria for the LBP and control groups are outlined in Table 1.

**TABLE 1:** Inclusion and exclusion criteria of patients with chronic low back pain and healthy controls.

Inclusion	Exclusion
Patients with chronic LBP	
Aged 18 or older Chronic low back pain (> 3 months) Willing and able to undergo lumbar spine MRI Sufficient English language proficiency to understand the study material, and capacity to consent	History of spine surgery Suspected or diagnosed malignancy, infection, or fracture Radiculopathy or claudication Pacemaker, implanted defibrillator, or other contraindications to MRI Greater than grade 1 spondylolisthesis, degenerative scoliosis, and severe facet arthropathy
Healthy controls	
Aged 18 or older No back pain in the previous 6 months Willing and able to undergo lumbar spine MRI Sufficient English language proficiency to understand the study material, and capacity to consent	History of spine surgery Radiculopathy or claudication Pacemaker, implanted defibrillator, or other contraindications to MRI

### 2.2 Data Collection

**FIGURE 1:**
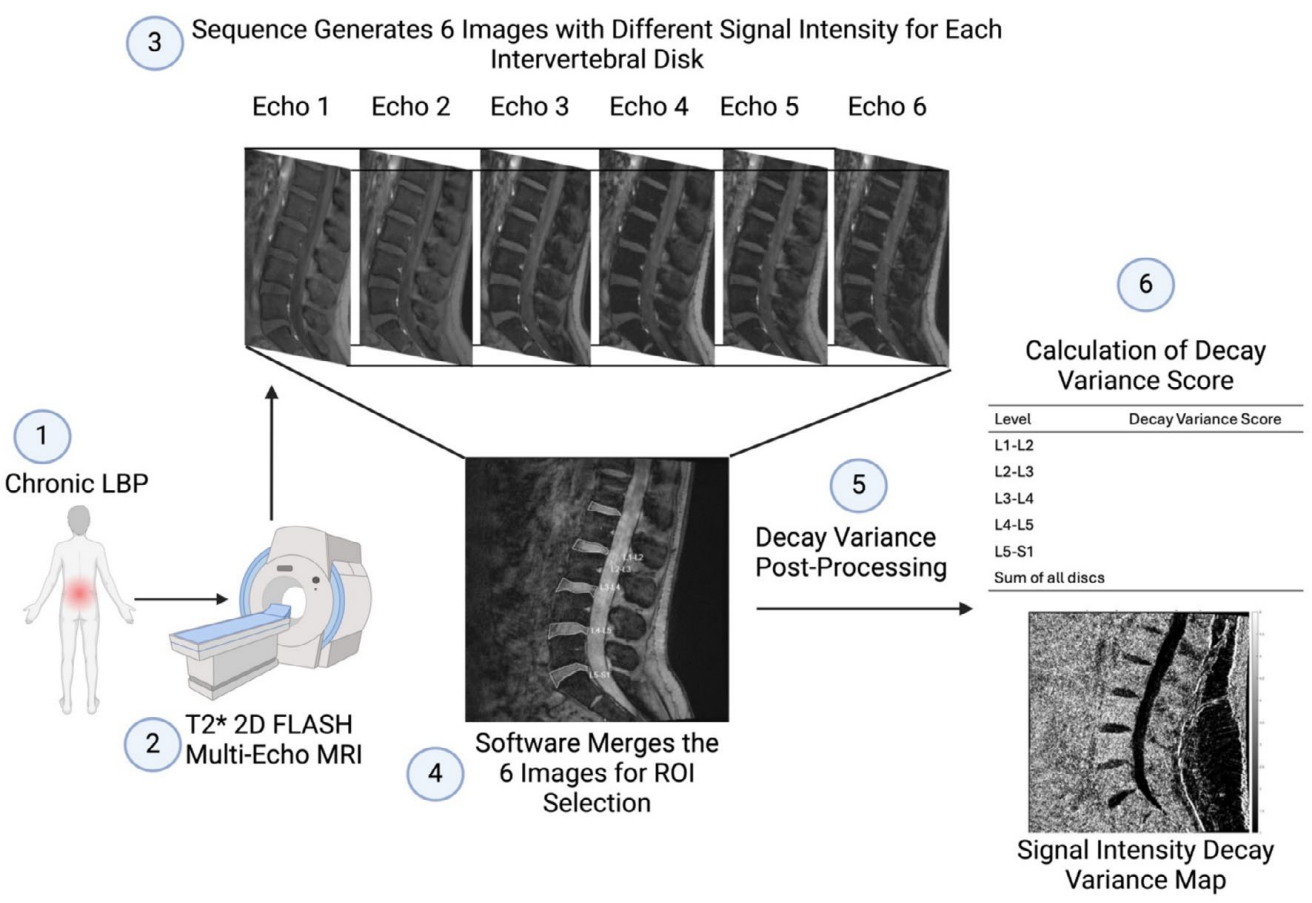
The Decay Variance (DeVa) workflow. (1) Patient with chronic low back pain is referred to a radiology centre for magnetic resonance imaging (MRI). (2) The patient undergoes a T2*‐weighted 2D FLASH multi‐echo MRI with a minimum of 6 echo times. (3) The sequence generates 6 images with different signal intensities for each intervertebral disc (IVD). (4) The DeVa software merges the 6 images into 1 in the working portal for the practitioner to trace each IVD as a region of interest (ROI). (5) The DeVa software then post‐processes the image. (6) A quantitative DeVa score for each disc as well as a qualitative signal intensity DeVa map is then generated in the form of a report (Appendix S2).

Participants recruited in the study were asked to rate their worst LBP and leg pain in the previous 7 days on an 11‐point (0–10) numerical rating scale (NRS) at the time of recruitment. Patients underwent MRI scanning on a 3 T Magnetom Lumina (Siemens) at a clinically accredited radiology practice in the same private hospital as the spine clinic in which recruitment occurred. Patients underwent a standard T2W MRI acquired in the sagittal and axial planes for the calculation of Pfirrmann grade and for determining whether stenosis, disc bulge, and high‐intensity zones were present in the IVD. A T2*‐weighted 2D FLASH (gradient echo) multi‐echo sequence, with a minimum of six echoes, was acquired in the mid‐sagittal plane. Six images with varying signal intensities were generated, which were subsequently merged into a single image by the software for post‐processing and DeVa score calculation. The DeVa workflow is summarized in Figure 1, key characteristics of the scan acquisition are provided in Table 2, and full anonymized DICOM header metadata are presented in Appendix S1.

**TABLE 2:** Key acquisition characteristics for the T2* 2D Flash sequence used for deriving processed decay variance scores for patient discs.

Characteristic	Value
Scanner type	Siemens Lumina
Field strength	3 T
Sequence	T2* 2D FLASH
Contrast mechanism	Gradient Echo
Plane	Midline sagittal
TE	2.94, 4.72, 6.50, 8.28, 10.06, 11.84 ms
TR	16 ms
Echo train length	6
Slice thickness	6 mm
Flip angle	25°
In‐plane resolution	1 mm isotropic

### 2.3 Decay Variance Postprocessing Calculation

Regions of interest (ROI) for each lumbar disc were created by manual segmentation of each IVD (defined as the NP and AF, excluding the EP) in the lumbar spine (including L5/S1) on a midline T2W MRI slice (confirmed via reference lines generated from an axial slice). For validation purposes, ROIs were created twice by two observers at least 1 week apart. All postprocessing was performed using Matlab (v. R2017b; Mathworks Inc., Natick, Massachusetts).

### 2.4 The DeVa Score of Each Pixel Was Calculated Using the Following Definitions

Signal Intensity (SI) recorded at the *i*th echo time *i* = 1, 2, …, *n*.

Signal Retention ratio (SR) at the (*i*+1)th echo time *i* = 1, 2, …, *n* − 1

$$SR_{i+1}=\frac{SI_{i+1}}{SI_{i}}$$

Signal Decay Change (SDC) at the (*i* + 2)th echo time i=1,2,…,n−2

$$\mathrm{SD}C_{i+2}=|SR_{i+2}-SR_{i+1}|$$

Thus, the formula for the Decay Variance method for n acquisitions can be represented as:

$$\mathrm{DV}=\sum_{i=1}^{n-2}\mathrm{SD}C_{i+2}$$

$$\mathrm{DV}=\sum_{i=1}^{n-2}|SR_{i+2}-SR_{i+1}|$$

$$\mathrm{DV}=\sum_{i=1}^{n-2}|\frac{SI_{i+2}}{SI_{i+1}}-\frac{SI_{i+1}}{SI_{i}}|$$

DeVa scores for each pixel were converted to disc‐wise scores by taking the arithmetic average of pixel scores in each ROI. A subsequent report was generated with the DeVa score and map (Appendix S2). These disc‐wise measures were converted into patient‐wise measures in two ways: firstly, by assigning to each patient the worst DeVa score measured in any of their lumbar discs (“Worst Disc”) and secondly by summating the measured scores for all six discs (“Total Discs”).

### 2.5 Statistical Analysis

The results were processed in two ways to address the aims of the study.

Firstly, to assess the relationship between degeneration and DeVa score, each lumbar IVD was dichotomized based on the presence of disc degeneration parameters (disc bulge, high‐intensity zones, and stenosis). The DeVa score for each individual disc was calculated, and an independent *t*‐test was performed to compare the DeVa score between groups. The Pearson correlation coefficient was employed to determine the association between the DeVa score and Pfirrmann grade (Table 3).

**TABLE 3:** Intraclass coefficients for DeVa scores and lumbar degenerative measurements.

Parameter	Intra‐rater analysis (ICC (95% CI))	Inter‐rater analysis (ICC (95% CI))
Decay Variance	0.960 (0.941, 0.973)	0.893 (0.845, 0.927)
Pfirrmann grade	0.927 (0.893, 0.950)	0.882 (0.830, 0.919)
Spinal stenosis	0.905 (0.862, 0.935)	0.823 (0.747, 0.877)
Foraminal stenosis	0.867 (0.808, 0.908)	0.929 (0.896, 0.951)
Disc bulge	0.909 (0.867, 0.938)	0.905 (0.862, 0.935)
High intensity zones	0.825 (0.750, 0.879)	0.830 (0.757, 0.882)

Secondly, participants' NRS scores were correlated to the “worst disc” and “total discs” DeVa score using a Pearson correlation coefficient. Participants were then dichotomized into “no pain” and “pain” subgroups. Comparison of average “worst disc” and “total discs” DeVa was presented as Gardiner‐Altman estimation plots of mean difference with effect size presented as Cohen's *d* with a bootstrapped confidence interval for effect size [18]. This analysis plan was developed with primacy of effect size measures over null hypothesis significance testing due to the strength of results from preclinical studies. However, *p*‐scores derived from an independent *t*‐test were also provided for completeness. Additionally, a subgroup analysis was performed to compare the DeVa score between patients with LBP and controls in participants with Pfirrmann grade ≤ 3 and no stenosis.

Statistical analyses were conducted using the commercially available software SPSS (version 27, IBM Corporation, New York, USA). The level of statistical significance was set at 5% (*p* = 0.05).

### 2.6 Reliability Analysis

Data points for 20 patients were measured by a second rater (A.S.) to evaluate inter‐rater reliability, and for a second time 3 weeks after initial extraction by the first author (S.S.) to evaluate intra‐rater reliability. To enhance the quality and applicability of this study, each rater was blinded to their own measurements and findings of the other. Inter‐rater reliability was assessed using the intraclass coefficient estimates (ICC) based on single‐rating, consistency, 2‐way random effects model, and intra‐rater reliability was assessed using ICC based on single‐rating, absolute agreement, 2‐way fixed effects model. ICC values of < 0.05, 0.5–0.75, 0.75–0.90, and > 0.90 indicated poor, moderate, good, and excellent reliability, respectively.

Both the intra‐ and inter‐rater reliability for DeVa score and lumbar degenerative parameters were good to excellent, from 0.823 (0.750, 0.879) to 0.960 (0.941, 0.973).

## 3 Results

### 3.1 Demographics

A total of 77 patients with chronic LBP and 8 control subjects were included in the study. The pain group's average Numerical Rating Scale (NRS) score was 7.9 ± 1.6. Patients with chronic LBP had a 42% and 22% higher DeVa score when using the worst disc (1.56 ± 0.48 vs. 1.10 ± 0.19, *p* < 0.01) and sum of all disc (5.68 ± 1.10 vs. 4.65 ± 0.61, *p* < 0.05) methods, respectively. No significant differences in age or sex were observed between the groups (Table 4).

**TABLE 4:** Patient demographics and clinical data.

Variable	Chronic LBP (*n* = 77)	Controls (*n* = 8)	*p*
Sex (M/F)	41/36	3/5	0.396
Age (years ± SD)	47.8 ± 15.8	43.8 ± 13.2	0.492
Pain severity (NRS ± SD)	7.9 ± 1.6		
DeVa score			
Worst disc	1.56 ± 0.48	1.10 ± 0.19	**< 0.01**
Sum of all disc	5.68 ± 1.10	4.65 ± 0.61	**< 0.05**

*Note:* *p*‐values in bold represent statistical significance (*p* < 0.05).

### 3.2 Correlation With Degeneration

**FIGURE 2:**
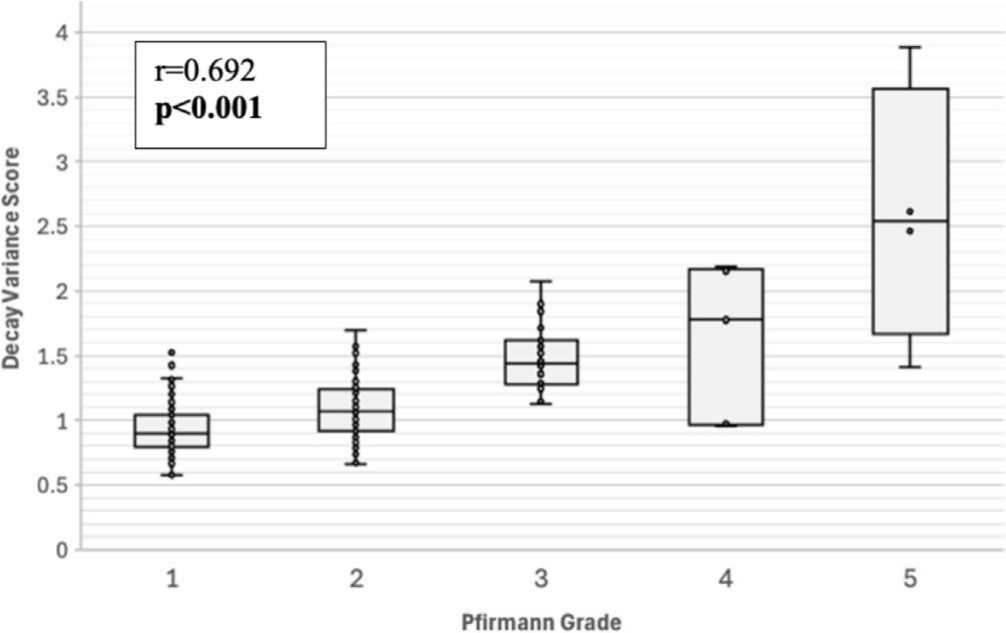
Association between Decay Variance score and Pfirrmann grade. A Pearson correlation coefficient demonstrated a strong positive association between Decay Variance score and Pfirrmann grade.

A strong positive correlation was observed between the DeVa score and Pfirrmann grade (*r* = 0.692, *p* < 0.001 Figure 2). Discs with stenosis (1.50 ± 0.64 vs. 1.08 ± 0.31, p < 0.001), disc bulge (1.35 ± 0.49 vs. 1.01 ± 0.24, *p* < 0.001), and high‐intensity zones (1.36 ± 0.37 vs. 1.09 ± 0.36, *p* < 0.001) exhibited significantly higher DeVa scores compared to discs without these features (Table 5).

**TABLE 5:** Association between Decay Variance score and Measures of Disc Degeneration.

Parameter	Yes	No	*p*
Stenosis			
*N*	40	385	
DeVa score	1.50 ± 0.64	1.08 ± 0.31	**< 0.001**
Disc bulge			
*N*	133	292	
DeVa score	1.35 ± 0.49	1.01 ± 0.24	**< 0.001**
High intensity zones			
*N*	46	379	
DeVa score	1.36 ± 0.37	1.09 ± 0.36	**< 0.001**

*Note:* *p*‐values in bold represent statistical significance (*p* < 0.05).

### 3.3 Correlation With Pain

**FIGURE 3:**
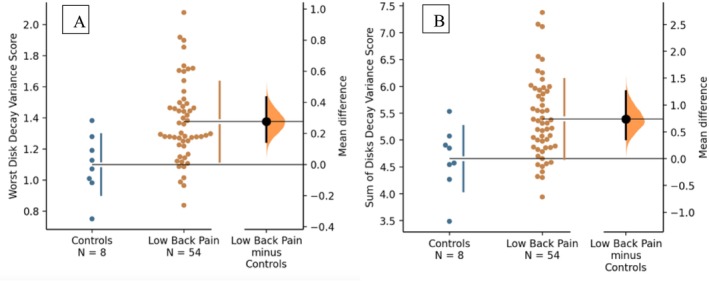
(A, B) Gardner‐Altman estimation plots demonstrating the difference in measured Decay Variance scores between participants with chronic LBP with Pfirrmann grade ≤ 3 and no stenosis (orange) with asymptomatic controls (blue). 2A and 2B demonstrate the difference in the worst disc and the sum of all discs decay variance score, respectively.

A moderate positive correlation was observed between the DeVa score and NRS using the worst disc (*r* = 0.296, *p* < 0.01) and the sum of all discs (*r* = 0.323, *p* < 0.005) methods. In a subgroup analysis of patients with a Pfirmann grade ≤ 3 and no stenosis (n=54), those with pain had a significantly higher DeVa score for the worst disc (1.38 ± 0.26 vs. 1.10 ± 0.29, Cohen's *d* = 1.1 [95% CI: 0.55, 1.7], *p* < 0.005, Figure 3A) and sum of all discs (5.39 ± 0.75 vs. 4.65 ± 0.61, Cohen's *d* = 1.01 [95% CI: 0.45, 1.7], *p* < 0.01, Figure 3B). Both measurements yielded a Cohen's *d* greater than 1, indicating a large effect size.

### 3.4 Qualitative Representation of Decay Variance Map

**FIGURE 4:**
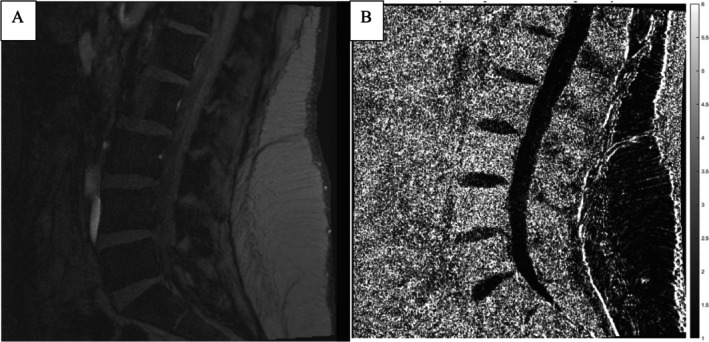
(A, B) This figure shows the same midline slice for a female patient in her fourth decade of life with chronic low back pain. (A) is a single slice of the underlying T2* 2D FLASH gradient echo sequence. (B) shows the calculated Decay Variance map. In this image, the L4/L5 disc has a heterogeneously increased Decay Variance compared to the other (healthy) discs with homogenously low Decay Variance.

The generated Decay Variance map also provides qualitative analysis of the discs. In the map, each pixel represents the summation of unsigned change in proportional signal loss between two consecutive pairs of echo times (sharing one mutual echo time) across all possible echo time triplets. Dark pixels indicate similar proportional signal loss between pairs of echo times (i.e., approximating an exponential decay curve) across all the echo times measured, whereas increasingly brighter pixels show increasing differences between proportional signal loss in consecutive pairs of echo times (i.e., greater deviation from a truly exponential curve). A homogenously low Decay Variance (i.e., a homogeneously dark disc) is representative of a healthy disc when compared to a heterogeneously increased Decay Variance (i.e., mixture of low and high signal intensity). Variance maps also show good differentiation between discs and vertebral bodies; however, this technique provides very poor tissue definition in structures outside the spine (Figure 4A,B).

## 4 Discussion

This study presents for the first time a human study of a novel T2* MRI post‐processing method, DeVa, demonstrating objective and quantitative discrimination in an imaging maker between healthy and degenerate discs as well as between patients with chronic LBP and asymptomatic controls.

One of the hallmarks of IVD degeneration is the progressive loss of extracellular matrix molecules, specifically the glycosaminoglycan‐substituted proteoglycans. This loss is often associated with increased extracellular catabolism via metalloproteinases and pro‐inflammatory cytokines, and decreased hydration of the IVD, which can lead to pain and further degeneration [19]. The findings of this study corroborate the biological rationale behind glycosaminoglycan dysregulation in disc degeneration, as a strong positive correlation was demonstrated between DeVa score and Pfirrmann grade, and IVDs with disc bulge, high intensity zones, and stenosis had a higher DeVa score compared to IVDs without. However, further in vitro histological and biological studies on human IVD specimens are required to confirm this causal relationship. Conventional MRI is limited in its ability to detect subtle variations in tissue structure and histology, often only identifying advanced stages of disc degeneration [20]. In contrast, DeVa offers a more sensitive and quantitative approach for assessing disc health. This technique can detect significant differences in disc degeneration, even in cases where conventional imaging modalities may fail to capture these nuanced changes in tissue structure.

Currently, LBP management faces two key diagnostic challenges: identifying patients with severe pain despite structurally normal IVDs and detecting asymptomatic individuals with degenerate IVDs [4]. A more sensitive measure of IVD degeneration is crucial for understanding the role of disc pathology in pain—since current methods struggle to pinpoint how disc degeneration really contributes to chronic LBP [21]. The DeVa technique regardless of whether patient DeVa scores were calculated as the total of all lumbar discs (representing global degeneration) of if only the worst disc was used, was able to differentiate patients with chronic LBP without severe signs of disc desiccation (Pfirrmann ≤ 3) and no stenosis from asymptomatic controls. Additionally, DeVa scores were moderately correlated with pain severity. This demonstrates that, in contrast to the findings of Corniola's team when examining Pfirrmann grades, there was a strong link between Decay Variance score and clinical severity in back pain sufferers [22].

While our findings demonstrated large effect sizes, with the majority of patients with LBP having DeVa scores that fell entirely outside the range observed in asymptomatic controls, some overlap was anticipated (Figure 2). This is likely attributable to the complex nature of LBP, which, according to the biopsychosocial model, is closely influenced by psychological factors and comorbid medical conditions [23, 24]. This study specifically validates a technique that measures the physical and biological aspects of disc degeneration, rather than the broader biopsychosocial factors reflected in the composite NRS for pain. These findings suggest that the DeVa technique may be especially valuable in identifying pain‐generating discs, even in cases of early or moderate degeneration where traditional grading systems might fail to detect significant pathology.

Our findings raise significant clinical implications for the management of chronic LBP with advanced imaging techniques. Current guidelines advise against routine imaging in LBP patients without red flags, due to the high prevalence of incidental findings in asymptomatic individuals [25, 26, 27]. Diagnosis of discogenic LBP is heavily reliant on invasive discography, although its clinical efficacy is highly debated. Studies have shown that discography can accelerate disc degeneration by inducing inflammatory responses and causing structural disruptions. This invasive procedure can exacerbate disc pathology, thereby raising concerns about its long‐term safety and utility [28].

In contrast, the DeVa technique offers a noninvasive, objective, and quantitative alternative for assessing disc health; it functions primarily as a measurement tool akin to a thermometer. DeVa correlates with histological findings, which are a tissue‐level outcome of molecular‐cellular‐level homeostasis or degeneration, providing a more precise assessment of tissue composition without the risks associated with procedures like discography [17]. This suggests that DeVa could fill a critical gap in current diagnostic paradigms, especially in cases where conventional imaging techniques provide insufficient information. Although DeVa differentiated asymptomatic controls from chronic LBP patients with minimal nuclear degeneration, it does not serve as a diagnostic tool for the pain‐generating disc. Our work indicates that the DeVa Score has greater sensitivity in picking up degeneration in the absence of structural changes of the spine associated with progressive degeneration; ideally in the traditional Pfirrmann grade 0–3 and potentially 4 scale. A lower Deva Score implies a non‐symptomatic non‐degenerate disc, while a higher score implies a more degenerate, possibly symptomatic disc. Ultimately, the clinical utility of DeVa should be for the clinician to determine when using DeVa alongside patient history, traditional imaging (X‐ray, MRI), and symptom presentation. Nonetheless, DeVa holds significant promise for improving clinical decision‐making in spine management. It has the potential to guide the use of non‐surgical interventions, such as regenerative biologics, and prevent unnecessary spinal fusions, thereby offering a safer and more targeted approach to managing chronic LBP [29].

Several limitations that impacted our results must be acknowledged. First, this was a cross‐sectional study, and as such, it is not possible to infer any longitudinal implications of DeVa scores in terms of disease progression or response to treatment. Future research should explore the prognostic utility of DeVa by following patients over time and assessing how DeVa changes with the progression of disc degeneration and clinical symptoms. Another important limitation of the study is the highly selected patient population. All patients had chronic LBP defined by strict NIH criteria, which may not represent the broader population of individuals suffering from non‐specific LBP. Most patients in our study had a pain score of at least 4 out of 10, which might have introduced an implicit severity threshold. As such, these findings may not be generalizable to patients with milder forms of LBP, who may not exhibit such pronounced differences in DeVa scores. Additionally, we did not include body mass index (BMI) as a variable in our analysis. Future studies validating this technique in larger populations will incorporate BMI as a variable to provide a more comprehensive analysis. Finally, the study was limited by a small sample size, particularly in the control group. Future research should expand the sample size and include a more diverse patient population from primary care settings to enhance the generalizability of findings, with the goal of developing a quantitative *Z*‐score diagnostic guideline for a healthy IVD.

## 5 Conclusion

The DeVa postprocessing technique is an effective marker of disc degeneration and pain. The DeVa score is strongly positively correlated with the Pfirrmann grade and is significantly higher in discs with stenosis, disc bulge, and high‐intensity zones. Clinically, the DeVa score is correlated with pain severity and effectively differentiates patients with chronic LBP who do not exhibit nuclear degeneration (Pfirrmann < 4) and stenosis from asymptomatic controls. Future research should include a larger control group and a more diverse patient population from primary care settings to validate the technique further. Ultimately, the DeVa technique represents a significant advancement in the field of spinal imaging. It offers a noninvasive, quantitative approach to assess degeneration and pain. This potentially opens new pathways for better selecting participants in trials of therapeutics aimed at IVD regeneration as well as provides information on culprit discs.

## 6 Disclaimer

This work was supported by a “Design and Innovation” grant of 45000CHF from AO Spine, Davos, Switzerland, a grant of $1 074 687.00 AUD through theMedical Research Future Fund (MRFF) BioMedTech Horizon (BMTH) program operated by MTPConnect, and a University Postgraduate Award from the University of New South Wales to S.S. and A.S. While D.A. and A.D. hold director's position in Merunova and J.K. has stocks in Merunova, which is a spin‐off that has rights to commercialize the associated technology, and the University of New South Wales may receive potential proceeds if and when that occurs, all other authors declare that they have no known competing financial interests or personal relationships that could have appeared to influence the work reported in this paper. Disclosures are in place in line with the UNSW's relevant policies. This study was also supported by Lumus Imaging for allowing us to use their MRI facilities, St George Private Hospital, and the Spine Service Clinical Trials Unit. Spine Labs is supported via unrestricted research grants to its institution by Baxter Inc.

## Conflicts of Interest

The authors declare no conflicts of interest.

## Supporting information


**Appendix S1.** Supporting Information.

**Appendix S2.** Supporting Information.

## References

[1] “Editorial, the Global Epidemic of Low Back Pain,” Lancet Rheumatology 5, no. 6 (2023): e305. 38251593 10.1016/S2665-9913(23)00133-9

[2] B.‐G. Peng , “Pathophysiology, Diagnosis, and Treatment of Discogenic Low Back Pain,” World Journal of Orthopedics 4, no. 2 (2013): 42. 23610750 10.5312/wjo.v4.i2.42 PMC3631950

[3] S. Sima and A. Diwan , “Contemporary Clinical Perspectives on Chronic Low Back Pain: The Biology, Mechanics, etc. Underpinning Clinical and Radiological Evaluation,” JOR Spine 8, no. 1 (2025): e70021. 39867670 10.1002/jsp2.70021 PMC11757297

[4] A. D. Diwan and J. Melrose , “Intervertebral Disc Degeneration and How it Leads to Low Back Pain,” JOR Spine 6, no. 1 (2023): e1231. 36994466 10.1002/jsp2.1231 PMC10041390

[5] S. Sima , X. Chen , and A. D. Diwan , “The Association Between Inflammatory Biomarkers and Low Back Disorder: A Systematic Review and Meta‐Analysis,” Biomarkers 29, no. 4 (2024): 171–184. 38578280 10.1080/1354750X.2024.2339285

[6] V. Tam , N. Chopra , S. Sima , et al., “Effects of GDF6 on Active Protein Synthesis by Cells of Degenerated Intervertebral Disc,” European Spine Journal (2025): ISSLS Prize Submissions, 1–13, 10.1007/s00586-025-08715-1. 39920317

[7] X. Chen , S. Sima , H. S. Sandhu , J. Kuan , and A. D. Diwan , “Radiographic Evaluation of Lumbar Intervertebral Disc Height Index: An Intra and Inter‐Rater Agreement and Reliability Study,” Journal of Clinical Neuroscience 103 (2022): 153–162. 35905524 10.1016/j.jocn.2022.07.018

[8] S. Sima , X. Chen , K. Sheldrick , J. Kuan , and A. D. Diwan , “Reconsidering High Intensity Zones: Its Role in Intervertebral Disk Degeneration and Low Back Pain,” European Spine Journal 33, no. 4 (2024): 1474–1483. 38381388 10.1007/s00586-024-08185-x

[9] S. Sima , S. Lapkin , and A. D. Diwan , “In Subjects With Chronic Low Back Pain, Does Neuropathia Exclusively Correlated to Neuronal Compression? A Correlation Study of PainDETECT Questionnaire and Corresponding MRI and X‐Ray Findings,” European Spine Journal 33, no. 4 (2024): 1465–1473. 38300298 10.1007/s00586-024-08156-2

[10] S. Lapkin , S. Sima , Z. Gan , and A. D. Diwan , “A Confirmatory Factor Analysis of an Electronic Format painDETECT Questionnaire for Patients With Low Back Pain,” Current Medical Research and Opinion 40, no. 2 (2024): 259–265. 38079336 10.1080/03007995.2023.2293570

[11] C. W. Pfirrmann , A. Metzdorf , M. Zanetti , J. Hodler , and N. Boos , “Magnetic Resonance Classification of Lumbar Intervertebral Disc Degeneration,” Spine 26, no. 17 (2001): 1873–1878. 11568697 10.1097/00007632-200109010-00011

[12] A. M. Ellingson , H. Mehta , D. W. Polly , J. Ellermann , and D. J. Nuckley , “Disc Degeneration Assessed by Quantitative T2*(T2 Star) Correlated With Functional Lumbar Mechanics,” Spine 38, no. 24 (2013): E1533–E1540. 23921323 10.1097/BRS.0b013e3182a59453 PMC3830665

[13] C. P. Paul , T. H. Smit , M. de Graaf , et al., “Quantitative MRI in Early Intervertebral Disc Degeneration: T1rho Correlates Better Than T2 and ADC With Biomechanics, Histology and Matrix Content,” PLoS One 13, no. 1 (2018): e0191442. 29381716 10.1371/journal.pone.0191442 PMC5790235

[14] A. M. Ellingson , T. M. Nagel , D. W. Polly , J. Ellermann , and D. J. Nuckley , “Quantitative T2*(T2 Star) Relaxation Times Predict Site Specific Proteoglycan Content and Residual Mechanics of the Intervertebral Disc Throughout Degeneration,” Journal of Orthopaedic Research 32, no. 8 (2014): 1083–1089. 24788830 10.1002/jor.22633 PMC4136382

[15] S. Hoppe , S. Quirbach , T. C. Mamisch , F. G. Krause , S. Werlen , and L. M. Benneker , “Axial T2* Mapping in Intervertebral Discs: A New Technique for Assessment of Intervertebral Disc Degeneration,” European Radiology 22 (2012): 2013–2019. 22544293 10.1007/s00330-012-2448-8

[16] K. Sheldrick , Decay Variance: A Novel Technique for the Diagnosis of Intervertebral Disc Degeneration (Faculty of Medicine, University of New South Wales, 2023).

[17] K. Sheldrick , U. Chamoli , K. Masuda , S. Miyazaki , K. Kato , and A. D. Diwan , “A Novel Magnetic Resonance Imaging Postprocessing Technique for the Assessment of Intervertebral Disc Degeneration—Correlation With Histological Grading in a Rabbit Disc Degeneration Model,” JOR Spine 2, no. 3 (2019): e1060. 31572977 10.1002/jsp2.1060 PMC6764792

[18] J. Ho , T. Tumkaya , S. Aryal , H. Choi , and A. Claridge‐Chang , “Moving Beyond P Values: Data Analysis With Estimation Graphics,” Nature Methods 16, no. 7 (2019): 565–566. 31217592 10.1038/s41592-019-0470-3

[19] E. S. Silagi , I. M. Shapiro , and M. V. Risbud , “Glycosaminoglycan Synthesis in the Nucleus Pulposus: Dysregulation and the Pathogenesis of Disc Degeneration,” Matrix Biology 71 (2018): 368–379. 29501510 10.1016/j.matbio.2018.02.025 PMC6119535

[20] L. M. Benneker , P. F. Heini , S. E. Anderson , M. Alini , and K. Ito , “Correlation of Radiographic and MRI Parameters to Morphological and Biochemical Assessment of Intervertebral Disc Degeneration,” European Spine Journal 14 (2005): 27–35. 15723249 10.1007/s00586-004-0759-4 PMC3476685

[21] K. Fujii , M. Yamazaki , J. D. Kang , et al., “Discogenic Back Pain: Literature Review of Definition, Diagnosis, and Treatment,” JBMR Plus 3, no. 5 (2019): e10180. 31131347 10.1002/jbm4.10180 PMC6524679

[22] M.‐V. Corniola , M. Stienen , H. Joswig , et al., “Correlation of Pain, Functional Impairment, and Health‐Related Quality of Life With Radiological Grading Scales of Lumbar Degenerative Disc Disease,” Acta Neurochirurgica 158 (2016): 499–505. 26783024 10.1007/s00701-015-2700-5

[23] S. Meints and R. Edwards , “Evaluating Psychosocial Contributions to Chronic Pain Outcomes,” Progress in Neuro‐Psychopharmacology & Biological Psychiatry 87 (2018): 168–182. 29408484 10.1016/j.pnpbp.2018.01.017 PMC6067990

[24] S. Sima , S. Lapkin , Z. Gan , and A. D. Diwan , “Association Between Non‐Spinal Comorbid Medical Conditions and Neuropathic Low Back Pain—A Further Unravelling of Pain Complexities in the Context of Back Pain,” Global Spine Journal (2024): 21925682241276441. 39133241 10.1177/21925682241276441 PMC11571500

[25] E. Al‐Hihi , C. Gibson , J. Lee , R. R. Mount , N. Irani , and C. McGowan , “Improving Appropriate Imaging for Non‐Specific Low Back Pain,” BMJ Open Quality 11, no. 1 (2022): e001539. 10.1136/bmjoq-2021-001539 PMC8862455 35190485

[26] M. Almeida , B. Saragiotto , B. Richards , and C. G. Maher , “Primary Care Management of Non‐Specific Low Back Pain: Key Messages From Recent Clinical Guidelines,” Medical Journal of Australia 208, no. 6 (2018): 272–275. 29614943 10.5694/mja17.01152

[27] B. W. Koes , M. Van Tulder , C.‐W. C. Lin , L. G. Macedo , J. McAuley , and C. Maher , “An Updated Overview of Clinical Guidelines for the Management of Non‐Specific Low Back Pain in Primary Care,” European Spine Journal 19 (2010): 2075–2094. 20602122 10.1007/s00586-010-1502-y PMC2997201

[28] E. J. Carragee , A. S. Don , E. L. Hurwitz , J. M. Cuellar , J. Carrino , and R. Herzog , “2009 ISSLS Prize Winner: Does Discography Cause Accelerated Progression of Degeneration Changes in the Lumbar Disc: A Ten‐Year Matched Cohort Study,” Spine 34, no. 21 (2009): 2338–2345. 19755936 10.1097/BRS.0b013e3181ab5432

[29] T. Hodgkinson , B. Shen , A. Diwan , J. A. Hoyland , and S. M. Richardson , “Therapeutic Potential of Growth Differentiation Factors in the Treatment of Degenerative Disc Diseases,” JOR Spine 2, no. 1 (2019): e1045. 31463459 10.1002/jsp2.1045 PMC6686806

