# Supplementary material for: DeVa (Decay Variance): A Novel Score Calculated via Postprocessing the Changes in Signal Intensity of an Intervertebral Disc in a T2* Multi‐Echo Magnetic Resonance Image Can Quantify Painful and Degenerate Lumbar Vertebral Discs
Source: JOR Spine. 2025 Mar 6;8(1):e70056. doi: 10.1002/jsp2.70056 (PMC11885163; doi:10.1002/jsp2.70056)
Supplement: Supplementary file 1 — Appendix S1. Supporting Information. [file JSP2-8-e70056-s002.pdf]

```
(0008,0005) SpecificCharacterSet CS # 10 1 [ISO_IR 100]
(0008,0008) ImageType CS # 38 6 [ORIGINAL\PRIMARY\M\NORM\DIS2D\MFSI
(0008,0012) InstanceCreationDate DA # 8 1 [20240419]
(0008,0013) InstanceCreationTime TM # 14 1 [190620.882500]
(0008,0016) SOPClassUID UI # 26 1 [1.2.840.10008.5.1.4.1.1.4]
(0008,0018) SOPInstanceUID UI # 58 1 [1.3.12.2.1107.5.2.50.175702.3000002
(0008,0020) StudyDate DA # 8 1 [20240419]
(0008,0021) SeriesDate DA # 8 1 [20240419]
(0008,0022) AcquisitionDate DA # 8 1 [20240419]
(0008,0023) ContentDate DA # 8 1 [20240419]
(0008,002a) AcquisitionDatetime DT # 22 1 [20240419190620.882500]
(0008,0030) StudyTime TM # 10 1 [185429.800]
(0008,0031) SeriesTime TM # 10 1 [190649.338]
(0008,0032) AcquisitionTime TM # 14 1 [190620.882500]
(0008,0033) ContentTime TM # 14 1 [190649.342000]
(0008,0050) AccessionNumber SH # 14 1 [91.45422306_1 ]
(0008,0060) Modality CS # 2 1 [MR]
(0008,0070) Manufacturer LO # 20 1 [Siemens Healthineers]
(0008,0080) InstitutionName LO # 26 1 [St George Private Hospital]
(0008,0081) InstitutionAddress ST # 46 1 [South Street 1,Kogarah,New South Wale
(0008,1010) StationName SH # 10 1 [AWP175702 ]
(0008,1030) StudyDescription LO # 28 1 [MRI LUMBAR SPINE - SCIATICA ]
(0008,1032) ProcedureCodeSequence SQ # 38 1
[
  (0008,0100) CodeValue SH # 6 1 [MRILSS]
]
(0008,103e) SeriesDescription LO # 28 1 [T2_STAR_ME_GRE_SAG_DR DIWAN ]
(0008,1040) InstitutionalDepartmentName LO # 4 1 [MRI ]
(0008,1050) PerformingPhysiciansName PN # 0 0 []
(0008,1070) OperatorsName PN # 0 0 []
(0008,1090) ManufacturerModelName LO # 14 1 [MAGNETOM Vida ]
(0008,1110) ReferencedStudySequence SQ # 8 0
[
]
(0008,1250) Undefined SQ # 368 1
[
  (0008,1140) ReferencedImageSequence SQ # 132 1
  [
    (0008,1150) ReferencedSOPClassUID UI # 28 1 [1.2.840.10008.5.1.4.1.1.4.1]
    (0008,1155) ReferencedSOPInstanc... UI # 54 1 [1.3.12.2.1107.5.2.50.175702.2024
    (0008,1160) ReferencedFrameNumber IS # 2 1 [1]
  ]
  (0020,000d) StudyInstanceUID UI # 34 1 [1.3.6.1.4.1.14301.91.4.45422306.1]
  (0020,000e) SeriesInstanceUID UI # 60 1 [1.3.12.2.1107.5.2.50.175702.2024041
```

```
    (0040,a170) PurposeOfReferenceCodeSequ... SQ   # 86      1
    [
        (0008,0100) CodeValue           SH   # 6       1      [121326]
        (0008,0102) CodingSchemeDesignator  SH   # 4       1      [DCM ]
        (0008,0104) CodeMeaning          LO   # 28      1      [Alternate SOP Class instance]
    ]
]
(0008,9206) VolumetricProperties            CS   # 6       1      [VOLUME]
(0018,0015) BodyPartExamined              CS   # 6       1      [LSPINE]
(0018,0020) ScanningSequence              CS   # 2       1      [GR]
(0018,0021) SequenceVariant               CS   # 2       1      [SK]
(0018,0022) ScanOptions                   CS   # 4       1      [PER]
(0018,0023) MRAcquisitionType             CS   # 2       1      [2D]
(0018,0024) SequenceName                  SH   # 6       1      [*fl2d6]
(0018,0025) AngioFlag                     CS   # 2       1      [N]
(0018,0050) SliceThickness                DS   # 4       1      [6.0]
(0018,0080) RepetitionTime                DS   # 4       1      [16.0]
(0018,0081) EchoTime                      DS   # 4       1      [2.94]
(0018,0083) NumberOfAverages              DS   # 4       1      [3.0]
(0018,0084) ImagingFrequency              DS   # 10      1      [123.25277]
(0018,0085) ImagedNucleus                 SH   # 2       1      [1H]
(0018,0087) MagneticFieldStrength         DS   # 4       1      [3.0]
(0018,0089) NumberOfPhaseEncodingSteps    IS   # 4       1      [288]
(0018,0091) EchoTrainLength               IS   # 2       1      [6]
(0018,0093) PercentSampling               DS   # 6       1      [100.0]
(0018,0094) PercentPhaseFieldOfView       DS   # 6       1      [100.0]
(0018,0095) PixelBandwidth                DS   # 6       1      [694.0]
(0018,1000) DeviceSerialNumber            LO   # 6       1      [175702]
(0018,1020) SoftwareVersion               LO   # 14      1      [syngo MR XA50 ]
(0018,1030) ProtocolName                  LO   # 28      1      [t2_star_me_gre_sag_Dr DIWAN ]
(0018,1080) BeatRejectionFlag             CS   # 2       1      [N]
(0018,1090) CardiacNumberOfImages         IS   # 2       1      [1]
(0018,1250) ReceivingCoil                 SH   # 12      1      [Spine_32_RS ]
(0018,1251) TransmittingCoil              SH   # 4       1      [Body]
(0018,1310) AcquisitionMatrix             US   # 8       4      [288\0\0\288]
(0018,1312) PhaseEncodingDirection        CS   # 4       1      [COL]
(0018,1314) FlipAngle                     DS   # 4       1      [25.0]
(0018,1316) SAR                           DS   # 10      1      [0.05839778]
(0018,5100) PatientPosition               CS   # 4       1      [HFS]
(0018,9004) ContentQualification          CS   # 8       1      [PRODUCT]
(0020,000d) StudyInstanceUID              UI   # 34      1      [1.3.6.1.4.1.14301.91.4.45422306.1]
(0020,000e) SeriesInstanceUID             UI   # 58      1      [1.3.12.2.1107.5.2.50.175702.300000[illegible]
(0020,0010) StudyID                       SH   # 14      1      [91.45422306_1 ]
(0020,0011) SeriesNumber                  IS   # 6       1      [11001]
```

(0020,0012) AcquisitionNumber IS # 2 1 [1]
(0020,0013) InstanceNumber IS # 2 1 [1]
(0020,0032) ImagePositionPatient DS # 26 3 [-3.82978\-69.4383\99.9044]
(0020,0037) ImageOrientationPatient DS # 52 6 [-1.24E-13\1.0\4.895E-12\0.025284
(0020,0052) FrameOfReferenceUID UI # 54 1 [1.3.12.2.1107.5.2.50.175702.2.202
(0020,0105) NumberOfTemporalPositions IS # 2 1 [1]
(0020,1040) PositionReferenceIndicator LO # 0 0 []
(0020,1041) SliceLocation DS # 8 1 [-1.30258]
(0020,9172) Undefined SQ # 132 1
[
(0008,1150) ReferencedSOPClassUID UI # 28 1 [1.2.840.10008.5.1.4.1.1.4.1]
(0008,1155) ReferencedSOPInstanceUID UI # 54 1 [1.3.12.2.1107.5.2.50.175702.20
(0008,1160) ReferencedFrameNumber IS # 2 1 [1]
]
(0021,0010) Proprietary Tag LO # 18 1 [SIEMENS MR SDS 01]
(0021,0011) Proprietary Tag LO # 18 1 [SIEMENS MR SDI 02]
(0021,0012) Proprietary Tag LO # 18 1 [SIEMENS MR SDR 01]
(0021,1001) Proprietary Tag IS # 2 1 [68]
(0021,1004) Proprietary Tag DS # 4 1 [1.0]
(0021,1005) Proprietary Tag IS # 8 3 [0\0\-10]
(0021,1006) Proprietary Tag LO # 4 1 [void]
(0021,1008) Proprietary Tag SH # 4 1 [Fast]
(0021,100a) Proprietary Tag DS # 4 1 [1.0]
(0021,100c) Proprietary Tag SH # 4 1 [+LPH]
(0021,100d) Proprietary Tag US # 2 1 [0]
(0021,100f) Proprietary Tag DS # 20 3 [30.312\28.138\29.305]
(0021,1010) Proprietary Tag IS # 8 1 [65010000]
(0021,1011) Proprietary Tag DS # 4 1 [0.0]
(0021,1012) Proprietary Tag FD # 8 1 [2.0]
(0021,1013) Proprietary Tag DS # 4 1 [0.0]
(0021,1014) Proprietary Tag IS # 2 1 [0]
(0021,1016) Proprietary Tag DS # 4 1 [0.0]
(0021,1017) Proprietary Tag DS # 4 1 [0.0]
(0021,1018) Proprietary Tag SH # 10 1 [Bore Local]
(0021,1019) Proprietary Tag OB # 104492 1 [3c\58\50\72\6f\74\6f\63\6f\6c\3e\20.
(0021,101b) Proprietary Tag DS # 4 1 [1.0]
(0021,101c) Proprietary Tag DS # 28 3 [0.0151522\22.4972\-0.0693696]
(0021,101d) Proprietary Tag IS # 2 1 [2]
(0021,1022) Proprietary Tag SH # 8 1 [SIEMENS]
(0021,1023) Proprietary Tag IS # 2 1 [0]
(0021,1025) Proprietary Tag SL # 12 3 [0\0\-1679]
(0021,1026) Proprietary Tag IS # 76 38 [0\0\0\0\0\0\0\0\0\0\0\0\0\0\0\0\0\0\0\0\
(0021,1027) Proprietary Tag US # 2 1 [1]
(0021,102a) Proprietary Tag IS # 32 11 [255\0\0\0\0\4885\0\4892\4893\0\0]

(0021,102b) Proprietary Tag	ST	# 28	1	[1;HFS;68;;F;0;2;-1412458878]
(0021,102c) Proprietary Tag	DS	# 24	3	[14400.0\685.343\685.343]
(0021,102d) Proprietary Tag	DS	# 22	3	[4.448\0.135068\1.95554]
(0021,102e) Proprietary Tag	IS	# 2	1	[2]
(0021,102f) Proprietary Tag	DS	# 14	3	[35.0\33.0\29.0]
(0021,1030) Proprietary Tag	DS	# 4	1	[0.0]
(0021,1031) Proprietary Tag	IS	# 6	1	[-1689]
(0021,1032) Proprietary Tag	SS	# 2	1	[0]
(0021,1033) Proprietary Tag	SH	# 4	1	[GC25]
(0021,1034) Proprietary Tag	DS	# 4	1	[1.0]
(0021,1035) Proprietary Tag	DS	# 8	1	[0.799531]
(0021,1036) Proprietary Tag	DS	# 4	1	[0.0]
(0021,1038) Proprietary Tag	DS	# 8	1	[523.478]
(0021,103b) Proprietary Tag	DS	# 4	1	[0.0]
(0021,103d) Proprietary Tag	CS	# 2	1	[2D]
(0021,1044) Proprietary Tag	DS	# 14	2	[100.0\0.42339]
(0021,1045) Proprietary Tag	CS	# 4	1	[YES]
(0021,1046) Proprietary Tag	DS	# 8	1	[4597.62]
(0021,1047) Proprietary Tag	CS	# 4	1	[IEC]
(0021,1050) Proprietary Tag	US	# 2	1	[0]
(0021,1051) Proprietary Tag	US	# 2	1	[1]
(0021,1053) Proprietary Tag	CS	# 2	1	[U]
(0021,105d) Proprietary Tag	SL	# 4	1	[-10]
(0021,105e) Proprietary Tag	LO	# 12	1	[FoV 220*220]
(0021,105f) Proprietary Tag	SH	# 6	1	[TP F10]
(0021,1060) Proprietary Tag	DT	# 22	1	[20240419190619.598566]
(0021,1061) Proprietary Tag	SH	# 2	1	[0]
(0021,1062) Proprietary Tag	FL	# 4	1	[103.751]
(0021,1103) Proprietary Tag	DS	# 8	1	[27632.5]
(0021,1104) Proprietary Tag	DS	# 4	1	[0.0]
(0021,1106) Proprietary Tag	LO	# 28	1	[X_1_1_1_1_1_1_1_1_1_1_1_740]
(0021,111a) Proprietary Tag	SH	# 8	1	[measured]
(0021,111c) Proprietary Tag	IS	# 2	1	[1]
(0021,1124) Proprietary Tag	IS	# 2	1	[0]
(0021,1133) Proprietary Tag	IS	# 4	1	[144]
(0021,1141) Proprietary Tag	SH	# 8	1	[measured]
(0021,1142) Proprietary Tag	IS	# 4	1	[2500]
(0021,1145) Proprietary Tag	SL	# 12	3	[0\0\-1689]
(0021,1148) Proprietary Tag	IS	# 2	1	[16]
(0021,1149) Proprietary Tag	IS	# 4	1	[288]
(0021,114f) Proprietary Tag	LO	# 6	1	[SP2-4]
(0021,1151) Proprietary Tag	UL	# 4	1	[134217729]
(0021,1158) Proprietary Tag	SH	# 8	1	[288*288s]
(0021,1159) Proprietary Tag	IS	# 8	3	[0\0\-10]

(0021,115b) Proprietary Tag FD # 24 3 [-3.8297844584412086\-69.43832874351

(0021,115e) Proprietary Tag IS # 2 1 [0]

(0021,1171) Proprietary Tag UT # 12 1 [XXXXXXXXXXXX]

(0021,1175) Proprietary Tag CS # 24 4 [ORIGINAL\PRIMARY\M\NONE]

(0021,1176) Proprietary Tag LO # 66 3 [ChannelMixing:ND=true_CMM=1_CDM=1

(0021,1177) Proprietary Tag LO # 6 1 [*fl2d6]

(0021,1178) Proprietary Tag CS # 6 1 [DIS2D]

(0021,1179) Proprietary Tag CS # 6 1 [DIS2D]

(0021,1188) Proprietary Tag DS # 8 1 [-1.30258]

(0021,118a) Proprietary Tag IS # 2 1 [1]

(0021,118e) Proprietary Tag ST # 48 1 [X_1_1_1_1_1_1_1_1_1_1_1_740_1_6_1_1_

(0021,1201) Proprietary Tag LO # 12 1 [ACQUISITION]

(0021,1202) Proprietary Tag LO # 12 1 [ACQUISITION]

(0028,0002) SamplesPerPixel US # 2 1 [1]

(0028,0004) PhotometricInterpretation CS # 12 1 [MONOCHROME2]

(0028,0010) Rows US # 2 1 [288]

(0028,0011) Columns US # 2 1 [288]

(0028,0030) PixelSpacing DS # 18 2 [0.763889\0.763889]

(0028,0100) BitsAllocated US # 2 1 [16]

(0028,0101) BitsStored US # 2 1 [12]

(0028,0102) HighBit US # 2 1 [11]

(0028,0103) PixelRepresentation US # 2 1 [0]

(0028,0106) SmallestImagePixelValue US # 2 1 [0]

(0028,0107) LargestImagePixelValue US # 2 1 [254]

(0028,0301) BurnedInAnnotation CS # 2 1 [NO]

(0028,1050) WindowCenter DS # 6 1 [180.0]

(0028,1051) WindowWidth DS # 6 1 [424.0]

(0028,1052) RescaleIntercept DS # 4 1 [0.0]

(0028,1053) RescaleSlope DS # 4 1 [1.0]

(0028,2110) LossyImageCompression CS # 2 1 [00]

(0029,0010) Proprietary Tag LO # 26 1 [INTELERAD MEDICAL SYSTEMS]

(0029,1010) Proprietary Tag SH # 10 1 [COMPRESSED]

(0029,1011) Proprietary Tag US # 2 1 [0]

(0029,1012) Proprietary Tag US # 2 1 [16]

(0029,1013) Proprietary Tag US # 2 1 [0]

(0029,1020) Proprietary Tag UN # 32 1 [e8fc77668bdaa136f6fbec6a819d19f7]

(0029,1021) Proprietary Tag US # 12 6 [1\5\10\90\95\99]

(0029,1022) Proprietary Tag FD # 48 6 [7.0001220703125\7.0001220703125\7.0

(0040,0009) ScheduledProcedureStepID SH # 14 1 [91.45422306_1]

(0040,0244) PerformedProcedureStepStartDate DA # 8 1 [20240419]

(0040,0245) PerformedProcedureStepStartTime TM # 14 1 [185429.800000]

(0040,0253) PerformedProcedureStepID SH # 16 1 [SIccc2c6cbdf5f4f]

(0040,0254) PerformedProcedureStepDescription LO # 28 1 [MRI Lumbar Spine - Sciatica

(0040,0275) RequestAttributesSequence SQ # 282 1

```
[
   (0008,0050) AccessionNumber          SH   # 14      1     [91.45422306_1 ]
   (0008,1110) ReferencedStudySequence     SQ   # 8       0
   [
   ]
   (0020,000d) StudyInstanceUID          UI   # 34      1     [1.3.6.1.4.1.14301.91.4.45422306.1]
   (0032,1060) RequestedProcedureDescription LO   # 28      1     [MRI Lumbar Spine - Sciatica ]
   (0032,1064) RequestedProcedureCodeSequ... SQ   # 38      1
   [
      (0008,0100) CodeValue          SH   # 6       1     [MRILSS]
   ]
   (0040,0007) ScheduledProcedureStepDesc... LO   # 28      1     [MRI Lumbar Spine - Sciatica ]
   (0040,0008) ScheduledActionItemCodeSeq... SQ   # 8       0
   [
   ]
   (0040,0009) ScheduledProcedureStepID    SH   # 14      1     [91.45422306_1 ]
   (0040,1001) RequestedProcedureID       SH   # 14      1     [91.45422306_1 ]
]
(0051,0010) Proprietary Tag             LO   # 18      1     [SIEMENS MR HEADER ]
(0051,1008) Proprietary Tag             CS   # 12      1     [IMAGE NUM X]
(0051,1009) Proprietary Tag             LO   # 4       1     [1.0 ]
(0051,100a) Proprietary Tag             LO   # 10      1     [TA: 27.63 ]
(0051,100c) Proprietary Tag             LO   # 12      1     [FoV 220*220 ]
(0051,100d) Proprietary Tag             SH   # 8       1     [SP R1.3 ]
(0051,1012) Proprietary Tag             SH   # 6       1     [TP F10]
(0051,1013) Proprietary Tag             SH   # 4       1     [+LPH]
(0400,0561) Undefined                 SQ   # 412     1
[
   (0400,0550) ModifiedAttributesSequence   SQ   # 266     1
   (0400,0562) Undefined              DT   # 18      1     [20240419190702.147]
   (0400,0563) Undefined              LO   # 44      1     [INTELEPACSPACS-5-3-1-P411LDSPACS-5-[illegible]
   (0400,0564) Undefined              LO   # 12      1     [sourceOfPrev]
   (0400,0565) Undefined              CS   # 8       1     [CORRECT]
]
(2050,0020) PresentationLUTShape          CS   # 8       1     [IDENTITY]
(3f01,0010) ImsPostFix                LO   # 26      1     [INTELERAD MEDICAL SYSTEMS ]
(3f01,1001) Proprietary Tag             LO   # 8       1     [NHSTGMR ]
(3f01,1003) Proprietary Tag             LO   # 10      1     [NHSTGMRMR1]
(3f01,1005) Proprietary Tag             LO   # 16      1     [AUHISNHSTGRGMOD2]
(3f01,1007) Proprietary Tag             SH   # 4       1     [true]
(3f01,1009) Proprietary Tag             LO   # 6       1     [AUHIS ]
(3f01,100a) Proprietary Tag             DA   # 8       1     [20240419]
(3f01,100b) Proprietary Tag             TM   # 6       1     [190610]
(3f01,1010) Proprietary Tag             LO   # 0       0     []
```

(3f01,1011)	Proprietary Tag	US	# 2	1	[256]
(3f01,1012)	Proprietary Tag	UL	# 4	1	[1713517570]
(3f01,1013)	Proprietary Tag	LO	# 22	1	[1.2.840.10008.1.2.4.90]
(3f01,1014)	Proprietary Tag	LO	# 16	1	[AUHISNHSTGRGMOD2]
(3f01,101d)	Proprietary Tag	LO	# 4	1	[597]
(3f03,0010)	Proprietary Tag	LO	# 26	1	[INTELERAD MEDICAL SYSTEMS]

404191908035960000247]

919061951839309101.0.0.0]

40419190803596000002451

24041919064931860809115]

[illegible]963\109.90443293050268]

\ACCAlgo:9\NormalizeAlgo...]

[illegible]001220703125\135.00207...]
