# Supplementary material for: DeVa (Decay Variance): A Novel Score Calculated via Postprocessing the Changes in Signal Intensity of an Intervertebral Disc in a T2* Multi‐Echo Magnetic Resonance Image Can Quantify Painful and Degenerate Lumbar Vertebral Discs
Source: JOR Spine. 2025 Mar 6;8(1):e70056. doi: 10.1002/jsp2.70056 (PMC11885163; doi:10.1002/jsp2.70056)
Supplement: Supplementary file 2 — Appendix S2. Supporting Information. [file JSP2-8-e70056-s001.pdf]

# Merunova

## Decay Variance Scan Results

Name:

Date of Birth:

Date of Scan:

This is a report of certain specific sequences of your lumbar spine MRI scan performed on {dd/mm/yyyy}. As such only represent one aspect of the MRI scan analysis and has to be read as a supplement to the radiologist report of the MRI. The focus of this report is to quantify the extent of disc degeneration in a narrow early stages of degeneration

## Decay Variance Results

| Level | Decay Variance Score | Z-score |
|---|---|---|
| L1-L2 | 0.869 | |
| L2-L3 | 0.892 | |
| L3-L4 | 1.09 | |
| L4-L5 | 1.30 | |
| L5-S1 | 0.900 | |

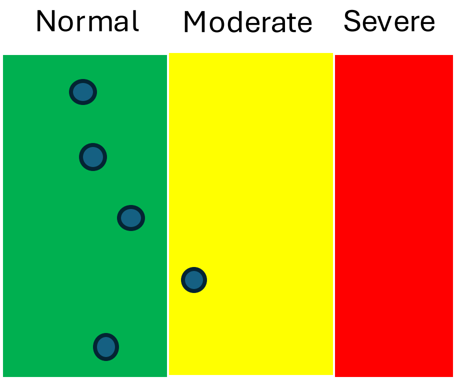


## Decay Variance Scan:

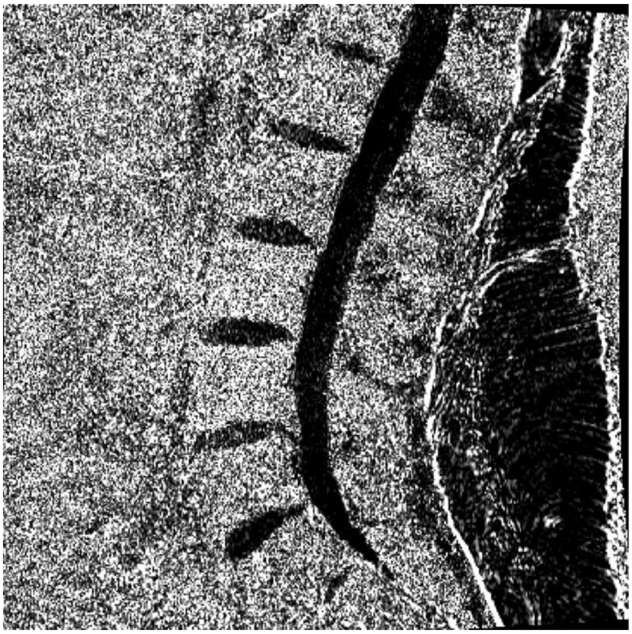

At this stage (2/25) the interpretation of the scores above are purely a reflection of the rate at which magnetic signal attenuate at the specific discs and rely on similar animal data to generate the degeneration score for specificity, as an alternative to Pfirrmann scores in use for research.

Your doctor/clinician will use this information to supplement your overall clinical pain profile, any xray or other evidence of progressive disc degeneration and any other diagnostic tests they use to identify a pain generator for your symptoms.

This report does not provide any diagnostic merit for tumors, metabolic disease or inflammatory arthritis.
